# *Chlamydiaceae*: Diseases in Primary Hosts and Zoonosis

**DOI:** 10.3390/microorganisms7050146

**Published:** 2019-05-24

**Authors:** Heng Choon Cheong, Chalystha Yie Qin Lee, Yi Ying Cheok, Grace Min Yi Tan, Chung Yeng Looi, Won Fen Wong

**Affiliations:** 1 Department of Medical Microbiology, Faculty of Medicine, University of Malaya, Kuala Lumpur 50603, Malaysia; hengchoon89@gmail.com (H.C.C.); chalystha@gmail.com (C.Y.Q.L.); heathercheok@gmail.com (Y.Y.C.); gtanmy89@gmail.com (G.M.Y.T.); 2 School of Bioscience, Taylor’s University, Subang Jaya 47500, Selangor, Malaysia; ChungYeng.Looi@taylors.edu.my

**Keywords:** *Chlamydiaceae*, *Chlamydia*, infection in primary host, zoonosis

## Abstract

Bacteria of the *Chlamydiaceae* family are a type of Gram-negative microorganism typified by their obligate intracellular lifestyle. The majority of the members in the *Chlamydiaceae* family are known pathogenic organisms that primarily infect the host mucosal surfaces in both humans and animals. For instance, *Chlamydia trachomatis* is a well-known etiological agent for ocular and genital sexually transmitted diseases, while *C. pneumoniae* has been implicated in community-acquired pneumonia in humans. Other chlamydial species such as *C. abortus*, *C. caviae*, *C. felis*, *C. muridarum*, *C. pecorum*, and *C. psittaci* are important pathogens that are associated with high morbidities in animals. Importantly, some of these animal pathogens have been recognized as zoonotic agents that pose a significant infectious threat to human health through cross-over transmission. The current review provides a succinct recapitulation of the characteristics as well as transmission for the previously established members of the *Chlamydiaceae* family and a number of other recently described chlamydial organisms.

## 1. Introduction

The *Chlamydiaceae* family comprises a group of Gram-negative, obligate intracellular microorganisms that have a propensity to infect the mucosal area, which can cause diseases in both humans and animals. A characteristic feature shared by all members of *Chlamydiaceae* is the conserved biphasic developmental cycle, which respectively alternates between the reticulate body and the elementary body, representing the stages of replication and infection. These organisms are also able to enter a persistent stage that acts as a coping strategy for many hostile conditions such as host immunity and nutrient deprivation, thus allowing their long-term survival inside the host cell [1]. The persistence and asymptomatic nature of *Chlamydiaceae* infection in humans often leads to underdiagnosis and delayed treatment, causing an increased global burden of chlamydial diseases. Unfortunately, there is no effective human preventive vaccine that exists to date.

Recent years have seen expansion in the family *Chlamydiaceae*. Today, this family includes 13 species belonging to the genus *Chlamydia*, namely *C. trachomatis*, *C. pneumoniae*, *C. abortus*, *C. caviae*, *C. felis*, *C. muridarum*, *C. pecorum*, *C. psittaci*, *C. suis*, *C. avium*, *C. gallinacea*, *C. serpentis*, and *C. poikilothermis*, as well as three members of the taxon *Candidatus*, i.e., *Ca. C. ibidis*, *Ca. C. corallus*, and *Ca. C. sanzinia* [2]. Certain species such as *C. trachomatis* and *C. pneumoniae* are well-described causative agents of female genital tract infection or trachoma, and respiratory tract infection, respectively. Other *Chlamydiaceae* such as *C. abortus*, *C. caviae*, *C. felis*, *C. pecorum*, *C. psittaci*, and *C. suis* infect animals as their primary host. Some of these species cause huge economic losses by infecting livestock, while others pose significant threats to humans due to their potential for zoonotic transmission. This review summarizes the characteristics and transmission for the known *Chlamydiaceae* (Table 1), including the newly reported species i.e., *C. avium*, *C. gallinacea*, *C. serpentis*, *C. poikilothermis*, *Ca. C. ibidis*, *Ca. C. corallus*, and *Ca. C. sanzinia.*

### 1.1. Chlamydia trachomatis

*C. trachomatis* predominantly infects the mucosal epithelia of the reproductive tract, leading to sexually transmitted disease in humans [56]. Genital *C. trachomatis* infection is among the most prevalent curable sexually transmitted infections (STIs) in the world, with approximately 131 million cases of infections occurring annually across the globe [90]. To date, a total of 19 distinct *C. trachomatis* serovars have been classified on the basis of antibody specificity toward the chlamydial major outer membrane protein (MOMP) with each exhibiting variable tissue tropism [91,92]. Serovars A, B, Ba, and C give rise to eye infection and represent the infectious cause of blinding trachoma, which is an ocular disease that is afflicting communities in impoverished regions with limited access to healthcare, and in the Middle East, Asia, and Africa in particular. Whereas serovars D, Da, E, F, G, Ga, H, I, Ia, J, and K are responsible for infections of the urogenital tract, the L-serovars (L1, L2, L2a, L3) invade the lymphatics and lymph nodes, resulting in lymphogranuloma venereum (LGV). LGV is more widespread in tropical areas in the world, including southeast Asia, India, West Indies, Africa, and South America [24,80,83,92,93,94]. However, outbreaks of LGV infection are being increasingly reported in the industrialized regions of Europe, Australia, and North America, chiefly affecting men who have sex with men who are often co-infected with human immunodeficiency virus (HIV) [93,95,96,97,98,99,100,101,102].

Despite being largely curable (≥97%) with an appropriate antibiotic regimen such as azithromycin and doxycycline [103,104], a high proportion of asymptomatic cases (50–70%) has presented an enormous hurdle to efforts aimed at controlling *C. trachomatis* infection. Control of the pathogen has been further complicated by the high incidence of reinfection; young working women, individuals with poor educational standards, as well as those with multiple sexual partners are amongst the groups at higher risk for reinfection [105]. Infection with *C. trachomatis* can result in a broad spectrum of urogenital tract pathologies. In females, the infection can cause mucopurulent cervicitis, urethritis, and salpingitis, while in males, the infection manifests clinically as non-gonococcal urethritis, prostatitis, epididymitis, and epididymis orchitis, as well as seminal vesiculitis [56,57,58,59,60]. In the absence of adequate treatment, localized infection from the cervix may ascend to the uterus and fallopian tubes, which could lead to the development of salpingitis or pelvic inflammatory disease (PID) [66,72]. *C. trachomatis* infection also increases the likelihood of coinfection with human papilloma virus (HPV), *Neisseria gonorrhoeae*, and HIV [106,107,108,109,110]. Perinatal exposure to *C. trachomatis* can result in conjunctivitis and lower respiratory tract infection in newborns [73,74]. The disease pathogenesis following *C. trachomatis* is attributable to the secretion of pathogen immunogenic molecules such as chlamydial protease-like activity factor (CPAF), which paralyzes the activity of neutrophils [111,112], virulence factors such as the 7.5-kb plasmid [113,114], and the ability of the pathogen to alter the proteome profile of the host cells [115,116].

Current available evidence suggest that *C. trachomatis* infection predicts the development of adverse long-term sequelae. Women with a single positive diagnosis of genital *C. trachomatis* have been shown to be 30% more likely to develop pelvic inflammatory disease (PID), ectopic pregnancy, and tubal factor infertility (TFI), and the risk of PID is increased by an additional 20% following multiple diagnoses of chlamydia infection [71]. Various other severe obstetrics and gynecological outcomes are associated with chlamydial infection such as preterm delivery, the premature rupture of membranes, and spontaneous abortion [67,68,69,70]. *C. trachomatis* is also an etiologic agent for Fitz-Hugh-Curtis syndrome [75,76,77]. Data from a meta-analysis study have shown a positive correlation between *C. trachomatis* infection and cervical cancer [78]. Separately, a serological study demonstrated that antibodies against *C. trachomatis* are related to a twofold higher risk of ovarian cancer [79]. *C. trachomatis* infection of the male genital tract has been found to be linked with reduced semen volume, apoptosis of spermatozoa, and sperm DNA fragmentation, suggesting the likely involvement of *C. trachomatis* in the induction of male infertility [61,62,63,64,65]. Beyond its connection to numerous pathologies of the human ocular and reproductive systems, *C. trachomatis* is recognized to be an infectious trigger for reactive arthritis, which is believed to arise through the dissemination of the bacteria to the joint causing persistent inflammation [34,35]. Although non-human models such as mice are currently being used in genital infection studies [117], *C. trachomatis* is considered an exclusive human pathogen, and there is no evidence to suggest that natural infection takes place in these animals [118].

### 1.2. Chlamydia pneumoniae

First established as a distinct species within the genus *Chlamydia* in 1989, *C. pneumoniae* is a common respiratory pathogen with a wide distribution around the globe [119,120]. Transmission of the bacterium occurs principally through the respiratory route without the involvement of an animal reservoir, although an alternate mode of transmission via contaminated surfaces has been suggested [121,122]. Infections with *C. pneumoniae* are mostly asymptomatic, but diseases can manifest as community-acquired pneumonia, chronic obstructive pulmonary disease, and pharyngitis [33,36,37,38,39,40,41,42]. The pathogen has also been implicated as a probable cause of asthma, primary biliary cirrhosis, atherosclerosis, and malignancy, and is known to be associated with the onset of reactive arthritis, although with a lesser frequency compared with *C. trachomatis* [34,35,43,44,45,46,47,48,49].

The global antibody prevalence for *C. pneumoniae* is high; it increases proportionately with age, in which the antibody rates climb steadily from two to nine years of age, reaching 50% by the age of 20, and peaking at 80% in males and 70% in females at old age [123]. Although humans are the primary reservoirs for infection, *C. pneumoniae* has been identified in non-human animals spanning from koalas, horses, and bandicoots to a wide range of reptiles encompassing snakes, iguanas, chameleons, frogs, and turtles [51,124,125]. Virtually all *C. pneumoniae* isolated from animals harbor a 7.5-kb plasmid that is similarly present in many other chlamydial organisms such as *C. trachomatis* and *C. muridarum*, which is absent in their human isolate counterparts [126]. It has been suggested that human *C. pneumoniae* strains may have originated in non-human animals, which have gradually adapted to human hosts through progressive loss in certain genes and plasmids, and ultimately sidestepping the requirement for animal reservoirs [127]. The clinical features associated with *C. pneumoniae* infection in animals are less well defined, but infected koalas have been shown to experience many of the signs of respiratory disease such as sneezing, coughing, chest congestion, difficulty in breathing, rhinitis, and nasal discharge [50]. Zoonosis has not been described among the animal *C. pneumoniae* isolates, but findings from earlier studies showing the presence of animal *C. pneumoniae* genotypes in humans suggest a potential cross-species transmission to humans [51,52].

### 1.3. Chlamydia abortus

*C. abortus* has garnered significant research attention owing to its potential to cause zoonotic infection, its veterinary importance, and its economic impact. To date, *C. abortus* has been found to infect a wide range of animals, and is significantly associated with enzootic abortions in ruminants. The bacterium has been detected in various animals such as goats, sheep, poultry, yaks, pigs, and farmed fur animals [128,129,130,131,132,133]. During infection, the pathogen targets the placenta, which leads to abortion in the later stages of gestation or the birth of weaker offspring if the pregnancy is bought to term [3]. Infectious abortion caused by *C. abortus* occurs during primary infection, but it does not affect subsequent pregnancies. Infection with *C. abortus* and subsequent abortion cases are mostly found in domestic ruminants such as sheep and goats [130,134]. Consequently, this pathogen has caused a major negative impact on the livestock industry for many countries around the world [119]. *C. abortus* is a well-documented zoonotic pathogen that most commonly affects pregnant women. Women who acquire the pathogen from exposure to infected tissues from small ruminants during pregnancy are at risk of abortion, stillbirth, and gestational septicaemia [4,5,8,9]. The extragestational infection of *C. abortus* manifested as PID has also been described [7]. More recently, atypical pneumonia related to *C. abortus* has been reported in Spain [6].

### 1.4. Chlamydia caviae

*C. caviae* is well-known for its ability to cause infection in guinea pigs. While infections in guinea pigs can be asymptomatic, clinical signs can present as mild to severe conjunctivitis with profuse serous to purulent ocular discharge sealing the eyelids. Diseases such as conjunctival chemosis, follicular hypertrophy, and pannus can develop shortly after infection with self-limiting keratoconjunctivitis [14,16]. Besides close contact transmission, the pathogen can be sexually transmitted, and the clinical course of urogenital tract infection in guinea pigs mimics many aspects of *C. trachomatis* infection in humans such as urethritis, cystitis, and ascending infection involving the fallopian tube and endometrium. Pups born from infected sows can acquire the bacteria, which leads to conjunctivitis [15]. *C. caviae* DNA have occasionally been found in cat, dogs, rabbits, and horses, suggesting the occurrence of natural infection, but the pathologies in these animals have not been clearly documented [14,135,136]. Few reported cases of zoonotic transmission related to *C. caviae* exist. In most instances, infections have been acquired from exposure to diseased guinea pigs [14,17], but a new report has surfaced recently outlining an incidence of infection with unknown origins of infection, although transmission through inadvertent contact with other animals cannot be ruled out [18]. These individuals reportedly experienced mild conjunctivitis and severe respiratory conditions due to community-acquired pneumonia, and therefore, the zoonotic capacity of *C. caviae* should not be underestimated [14,17,18].

### 1.5. Chlamydia felis

Exposure to *C. felis* results in the development of conjunctivitis in cats, usually with minimal respiratory signs. Early features of infection in cats typically present as unilateral ocular disease, which may progressively develop toward bilateral conjunctivitis accompanied by hyperaemia of the nictitating membrane, blepharospasm, ocular discomfort and discharges, and conjunctival chemosis [20]. The pathogen transmits among cats through direct contact with infectious materials, specifically ocular secretions. Experimental infection of the genital tract in cats with *C. felis* developed chronic salpingitis with ensuing oviduct infection [19]. *C. felis* can be recovered from the vagina and rectum of cats, but the role of the sexual route in the transmission of the pathogen is currently unclear [19,20]. Seroprevalence for *C. felis* is relatively high in many countries, including China, Italy, Japan, and Slovakia, particularly among stray (>10%) and house cats (>3%) [137,138,139,140,141]. Although the bacterium is mainly carried by cats, dogs have also been reported to be an important reservoir of *C. felis* [137]. Therefore, the ubiquity of cats and dogs and their interactions with humans may facilitate the dissemination of *C. felis* to humans [137,138,139,140,141]. Indeed, a previous seroepidemiological study in Japan found that 1.7% of the general populace and 8.8% of small animal clinics’ veterinarians showed antibody prevalence toward *C. felis* Fe/Pn1 [141]. Despite this, evidence linking *C. felis* to severe diseases in humans is ambiguous [21]. Conjunctivitis in human has previously been found in a case report involving an HIV-positive patient whose infection was traced back to a personal pet kitten which tested positive for non-*trachomatis Chlamydia* [142]. A similar case was recently reported in a woman who contracted unilateral chronic conjunctivitis from her *C. felis*-positive kitten [143]. Cases such as these are rare, but contribute to the increased likelihood of a threat of zoonosis.

### 1.6. Chlamydia muridarum

*C. muridarum* (mouse pneumonitis agent) is a rodent pathogen that most commonly infects mice but may occasionally be found in chickens [22,25]. Two strains of *C. muridarum* that are currently known are Nigg and Weiss isolates, which have differing virulence and growth characteristics. The *C. muridarum* Nigg isolate forms bigger inclusions in vitro compared to the Weiss isolate, but the latter shows a higher virulence in vivo through intravaginal or respiratory infection [144]. Although the reproductive tract of rodents is not a natural site of chlamydial infection, *C. muridarum* can cause pathology in mice that is hormonally manipulated through progesterone injection a few days prior to intravaginal inoculation with high doses of bacteria. This approach of *C. muridarum* infection in mice is extensively employed as a model to study *C. trachomatis* infection, as many of its pathologies closely correlate with human chlamydiosis; including cervicovaginal infection, oviduct occlusion, and hydrosalpinx formation in female mice [24,25]. The acute phase of an infection of *C. muridarum* in female mice usually takes 30 days to resolve, and symptoms such as hydrosalpinx often appear in some infected mice two to three months later. The *C. muridarum*-infected mice are later immune to subsequent reinfection of the same pathogen after the disease is resolved [145,146]. *C. muridarum* infection in male mice causes urethritis without impairing male infertility or sperm quality [147].

### 1.7. Chlamydia pecorum

*C. pecorum* is a pathogen contributing to substantial koala population decline. *C. pecorum* is hyperendemic among koalas with prevalence estimates ranging from 50–90%, and remain asymptomatic in most cases [148,149]. The venereal route is considered to be the primary mode of dissemination of this pathogen among the koalas; alternate means of transmission via pap feeding, whereby the koala joeys consume the maternal fecal material, also may be possible [148]. In the koalas, infections caused by *C. pecorum* are linked to devastating outcomes including pneumonia, ocular infections (conjunctivitis and blindness), inapparent intestinal infection, and infections of the urinary as well as reproductive tracts, which can cause incontinence, cystitis, nephritis, and infertility [27]. In addition to koalas, *C. pecorum* infects other free-ranging and domestic species such as sheep, cattle, water buffalos, swine, bandicoots, pigeons, and recently, the infection of semi-domesticated reindeer has also been reported [150,151]. The clinical diseases in these animals are mostly similar to those in koalas, and may be accompanied by other manifestations such as polyarthritis, sporadic bovine encephalomyelitis, and enteritis [26,31,32]. Several reports have found a possible association between *C. pecorum* and abortion in ruminants such as buffalos, goats, and ewes [28,29,30]. Nonetheless, the zoonotic risk associated with *C. pecorum* is as yet unknown.

### 1.8. Chlamydia psittaci

*C. psittaci* is an important causative agent of widespread zoonotic psittacosis, which is otherwise known as ornithosis or parrot fever. The pathogen primarily infects birds, and could be disseminated to other organisms, including humans through the respiratory tract infection [53]. The serotyping method using monoclonal antibodies against the MOMP reveals a total of six avian (A–F) and two mammalian (WC and M56) serotypes, each with distinct levels of host specificity. Serotype A is mainly isolated from psittacine birds, B is primarily isolated from pigeons, C is mainly isolated from ducks and geese, D is mainly isolated from turkeys, E is mainly isolated from pigeons and other avian species, and F is mainly isolated from parakeets and turkeys [152,153]. Molecular genotyping targeting the *OmpA* gene encoding the MOMP reveals additional genotypes including E/B, which is found in pigeons, along with types I and J, which have high genetic similarity with the *C. psittaci* genotype F and *C. abortus*, respectively [154,155].

In psittacine birds, the parents can pass the infection to their offspring via regurgitation, thus causing chronic chlamydiosis. Symptoms of bird infection can include conjunctivitis, rhinitis, and blepharitis. Infected birds can spread the bacteria through fecal or nasal discharges, which poses serious risk for zoonotic transmission through the inhalation of infectious air droplets or dust particles [53,54]. This leads to symptoms in humans such as fever, chills, headache, myalgia, and malaise with or without respiratory symptoms [55]. Its ability for airborne transmission has led to the United States Center for Disease Control and Prevention (CDC) to classify *C. psittaci* as Category B Crucial Biological agents that may be potentially misused as a biological warfare agent [156]. In fact, a pandemic with more than 700 cases of human psittacosis worldwide have been reported to be associated to the large-scale shipment of infected parrots from Argentina between 1929–1930 [157]. This aside, other reported outbreaks are smaller and rare, such as a recent incident reported in France with eight women infected from the gutting and handling of infected chickens in 2013 [158].

### 1.9. Chlamydia suis

*C. suis* is the most prevalent *Chlamydiaceae* found in porcine population that causes diseases ranging from asymptomatic to mild respiratory infections [84], conjunctivitis [85], enteritis [86], and reproductive failure [159]. Currently, porcine are the only known natural host. The zoonotic transmission of *C. suis* has been described among farmers in the porcine slaughterhouse through screening, although there is no clear signs of a symptomatic infection [87,88,89,142,143]. The isolation of a tetracycline-resistant strain of *C. suis* has raised considerable concerns within the porcine farming industry, especially regarding the fear of horizontal transfer of the tetracycline resistant *Tet*(C) gene to other human chlamydial pathogens [160]. *C. suis* is the first obligate intracellular organism that has been shown to develop antibiotic resistance by horizontal gene transfer. The isolation of tetracycline-resistant strains of *C. suis* has been described since 1998 from infected swine in the US [161], and later in several European countries [162,163,164]. This is mainly a consequence of the antibiotics that are used in intensive farming industries and inadequate treatment of the infection [165].

### 1.10. Other Chlamydiaceae spp.

*C. avium*, *C. gallinacea*, and *Ca. C. ibidis* are three newly described avian chlamydial species. Thus far, *C. avium* has been detected in pigeons and psittacine birds, whereas *C. gallinacea* is endemic in domestic poultry, and is capable of infecting chickens, ducks, guinea fowls, turkeys, and possibly other birds [12,13,22,23,158,166,167,168,169,170,171]. Thus far, feral sacred ibis is the sole known animal reservoir for *Ca. C. ibidis* [172]. The pathogenicity and pathology of these chlamydial species have not been studied. Limited evidence suggests that *C. avium* and *C. gallinacea* may be able to cause respiratory disease in psittacine birds and pigeons, as well as reduced body weight in chickens, respectively [10,11,12,13,22]. All three avian chlamydial agents are not known to be pathogenic for humans. Despite this, a zoonotic potential has been suggested for *C. gallinacea* in France, where several incidences of atypical pneumoniae were reported among slaughterhouse workers [13,23]. On the other hand, *C. serpentis*, *C. poikilothermis*, *Ca. C. corallus*, and *Ca. C. sanzinia* are chlamydial organisms found in captive snakes. The host range for these chlamydial isolates has yet to be defined, and snakes are currently the only known animals harboring these organisms. Little is presently understood about the pathogenicity potentials of these bacteria, as no pathologies assigned to these species have been described in animals and humans [173,174,175].

## 2. Conclusions

An overview of the different types of *Chlamydiaceae* enables a better understanding of the different pathogenesis of the bacteria in the primary host and human. Most of the diseases caused by the *Chlamydiaceae* species in their primary host resemble the features in human diseases, which could serve as a model for understanding the transmission route, pathogenesis, and development of therapeutic and vaccination strategies. The study of *Chlamydiaceae* in different hosts is essential, as there is gaining concern on issues of public health such as antibiotic resistance through the horizontal gene transfer mechanism among the bacteria; perhaps, the intensive use of antibiotics in farming industries need to be controlled to curb the problem. Finally, although several new species have been reported, any interpretation must be cautious, as some of the classification depends merely on the differences in sequences, which are considered as minor and inadequate [176].

## Figures and Tables

**Table 1:** The list of members in the *Chlamydiaceae* family, and the diseases caused by each species in their primary host and human. PID: pelvic inflammatory disease.

Species	Primary Host	Diseases in Primary Host	Transmission to Human
*C. abortus*	Small ruminants e.g., sheep and goats	Abortion in late gestation or deliver weak/dead fetus [3]	Possible though close contact with infected tissues, causes abortion, stillbirth, gestational septicaemia, PID, and atypical pneumonia [4,5,6,7,8,9]
*C. avium*	Avian e.g., pigeons and psittacine birds	Respiratory disease in psittacine birds and pigeons [10,11,12,13]	Unknown
*C. caviae*	Guinea pigs, cat, dogs, rabbits, and horses	Conjunctivitis and urogenital tract infections [14,15,16]	Possible though close contact, causes mild conjunctivitis, severe community-acquired pneumonia [14,17,18]
*Ca. C. corallus*	Snakes	Unknown	Unknown
*C. felis*	Felines, especially cats, and dogs	Conjunctivitis with minimal respiratory disease, and upper reproductive tract infections [19,20]	Possible cause of conjunctivitis in human [21]
*C. gallinacea*	Domestic poultry e.g., chickens, ducks, guinea fowls, turkeys	Reduced body weight [22]	Possible cause of atypical pneumoniae [13,23]
*Ca. C. ibidis*	Feral sacred ibis	Unknown	Unknown
*C. muridarum*	Rodents e.g., mouse, and chickens	Cervicovaginal infection, oviduct occlusion, hydrosalpinx formation in female mice [24,25]	-
*C. pecorum*	Koala, livestock species including cattle, sheep, goats, water buffalos, swine, bandicoots, and pigeons	Pneumonia, conjunctivitis, blindness, urinary incontinence, cystitis, nephritis, abortion, infertility, polyarthritis, sporadic bovine encephalomyelitis, and enteritis [26,27,28,29,30,31,32]	Unknown
*C. pneumoniae*	Human and a wide range of non-human mammals and reptiles encompassing koalas, horses, bandicoots, snakes, iguanas, chameleons, frogs, and turtles	Humans: Community-acquired pneumonia, reactive arthritis, chronic obstructive pulmonary disease, and pharyngitis [33,34,35,36,37,38,39,40,41,42] Implicated in the onset and progression of asthma, primary biliary cirrhosis, atherosclerosis, reactive arthritis, and lung cancer [34,35,43,44,45,46,47,48,49] Animals: Largely undescribed. Infected koalas exhibit signs related to respiratory disease that encompass sneezing, coughing, chest congestion, difficulty in breathing, rhinitis, as well as nasal discharge [50]	Unknown. However, the discovery of animal genotypes of *C. pneumoniae* in humans suggests a likelihood for zoonotic transmission [51,52]
*Ca. C. sanzinia*	Snake	Unknown	Unknown
*C. psittaci*	Avian	Psittacosis/ornithosis conjunctivitis, rhinitis, and blepharitis [53,54]	Possible through inhalation; causes fever, chills, headache, myalgia, and malaise with or without respiratory symptoms [55]
*C. trachomatis*	Human	Males: Non-gonococcal urethritis, prostatitis, epididymitis, epididymis orchitis, as well as seminal vesiculitis [56,57,58,59,60]. Likely role in infertility due to evidence of reduced semen volume, apoptosis of spermatozoa, and sperm DNA fragmentation following infection [61,62,63,64,65] Females: Mucopurulent cervicitis, urethritis, and salpingitis [56,59,60]. Adverse obstetrics and gynecological complications including salpingitis or PID, ectopic pregnancy, tubal factor infertility (TFI), preterm delivery, premature rupture of membranes, and spontaneous abortion [66,67,68,69,70,71,72]. Exposure of infants to the bacterium can cause conjunctivitis and lower respiratory tract infection in newborns [73,74]. Fitz-Hugh-Curtis syndrome [75,76,77]. Cervical and ovarian cancer [78,79]. Both sexes: Trachoma [80,81] Lymphogranuloma venereum (LGV) [82,83] Reactive arthritis [34,35]	-
*C. serpentis*	Snakes	Unknown	Unknown
*C. suis*	Swine	Respiratory disorders [84], conjunctivitis [85], enteritis [86], and reproductive failure	Possible through close contact, no reported symptoms [87,88,89]
